# Switching for the better? understanding how contextual conditions influence patients’ choice of primary care provider

**DOI:** 10.1186/s12913-026-15798-z

**Published:** 2026-10-11

**Authors:** Caroline Blomberg-Ohlström, Inger K Holmström, Magnus Fredriksson, Ulrika Winblad

**Affiliations:** 1 Department of Public Health and Caring Sciences, Health Services Research, Uppsala University, Box 564, Uppsala, 751 22 Sweden; 2 Department of Journalism, Media and Communication, University of Gothenburg, Box 710, Gothenburg, 40530 Sweden; 3 Department of Health Sciences, Innovation and Design, Mälardalen University, Box 883, Västerås, 721 23 Sweden

**Keywords:** Primary care, Informed choice, Contextual conditions, Sweden, Thematic analysis

## Abstract

**Background:**

Considering current expectations that patients will stimulate competition based on quality among healthcare providers through informed choices, this study aims to advance understanding of patients’ limited engagement in choosing a primary care provider.

**Methods:**

The study focuses on how contextual conditions—such as institutional and social settings—influence decision-making. Conducted in a Swedish primary care context, the study employed a qualitative approach, using thematic analysis of interviews with 18 respondents who had experience seeking primary care services. To further explore contextual influences, respondents were asked to reflect on and contrast their decisions regarding primary care providers with those related to other services, such as nursing homes or schools.

**Results:**

Respondents were generally less engaged in seeking information about primary care providers compared to other types of service choices. Five themes of contextual conditions were found to influence engagement in the informed choice process: (1) the perceived necessity to choose, (2) the visibility of differences between providers, (3) the expectation of equal service quality, (4) the presence of personal relationships connected to providers, and (5) the continuity of the care relationship.

**Conclusions:**

Findings suggest that efforts to promote informed patient choice in primary care need to extend beyond the provision of information to address the contextual conditions that shape engagement. Policymakers aiming to increase competition and patient agency in healthcare should make provider differences more visible and clarify the purpose and impact of choice.

**Clinical trial registration:**

Not relevant.

## Background

Choice of health care provider has become an influential steering logic of the organization of health care services [1, 2]. An emphasis on choice of provider conveys principles like a conventional market where patients make active choices based on information about different providers’ services and clinical quality [3, 4]. In theory, patients’ choice of health care provider would constitute a feedback model, signaling to providers how they could tailor the range and quality of services to patients’ preferences. This, in turn, could lead to increased competition, quality, and efficiency throughout the health system [5, 6]. Yet, results from previous studies show that few patients are willing to consider different alternatives prior to choice [7, 8] and that they often overlook the type of information which could guide them in selecting the best-performing provider in terms of, for instance, competence and accessibility [9, 10]. This lack of engagement is perplexing, as an uninformed choice may result in care that is not aligned with patients’ preferences or, at worst, have adverse effects on their health and well-being. Ultimately, it challenges the realization of expected benefits from increased competition.

Hence, the overall aim of this study is to advance understanding of why patients show limited involvement in making informed choices of health care providers, specifically when choosing a primary care provider. Patients’ engagement in information-seeking may be shaped not only by individual factors but also by contextual conditions surrounding the choice, including social, economic, institutional, political, and geographical factors [11, 12]. These conditions can influence both the opportunities and constraints associated with making a choice, including the social norms and expectations surrounding the choice, as well as the regulatory and economic contexts in which the choice is made. To this end, we examine how patients understand the contextual conditions surrounding the choice of primary care provider in comparison with other public and private services, such as education, elderly care, and mobile operators. By comparing these choice contexts, we seek to identify contextual conditions that may distinguish primary care choice from other choice situations and explore how such differences may be associated with patients’ inclination to seek and use information.

The paper is organized as follows: first, an overview of previous literature in the field, which outlines the rationale for conducting the study and briefly explains the research approach. This section also introduces the specific research questions to be investigated. Second, the study setting is presented, followed by a more detailed description of the research design as well as a methods section. Third, the main findings are presented, followed by a discussion of the results and methodological considerations. A conclusion section, which outlines the major theoretical and policy contributions closes the study.

### Knowledge gap and study rationale

Evidence suggests that healthcare providers’ quality may influence patients’ choices, although its influence appears modest compared with more readily observable factors such as geographical distance, and may not always translate into actual provider choices [13, 14].

Previous research has largely focused on how patients’ internal, cognitive abilities and constructions limit their engagement in informed choice. Results demonstrate that patients who are presented with information about the quality of health care providers are often overwhelmed by its complexity and may struggle to process and use the information effectively due to limited health literacy, cognitive constraints or psychosocial factors [15–17]. Patients may also perceive that the information available to support provider choice does not adequately reflect the aspects of care they consider most important [18]. Recent qualitative research suggests that patients take a broader range of characteristics into account when choosing a primary care provider, such as communication, interpersonal qualities, and convenience, aspects that are only partially captured by conventional performance measures [19].

Earlier studies emphasizing patients’ cognitive barriers to processing information, and on whether available information is appropriately designed and relevant to patients’ preferences tend to assume that patients will automatically seek and engage with such information when faced with a choice. Newer research has therefore increasingly examined what motivates patients to consider or to seek information prior to the choice in the first place. Findings demonstrate that patients’ internal constructions of the choice situation partially explain their information-seeking practices. Specifically, patients who are dissatisfied with their current provider or believe that another provider offers superior healthcare quality are more likely to seek information before making a choice [20]. More recent research similarly shows that even patients who actively seek information tend to rely on basic information and partisan sources rather than information about clinical quality [9]. However, subsequent research has not confirmed that the group of active information seekers also seeks the types of information or uses the information sources that have the potential to support the choice of the best-performing providers. Hence, internal conditions, those originating within the individual, do not appear to fully explain what drives patients’ information practices when choosing a health care provider. This knowledge gap prompts the need to examine how external conditions, here referred to as contextual conditions, may influence the extent to which patients engage in information-seeking prior to making a choice. Drawing on research highlighting the role of contextual factors in health decision-making and participation in health-care decision processes [11, 12], we conceptualize contextual conditions as factors external to the individual that shape the circumstances under which a choice is made and may, consequently, influence patients’ inclination to engage in an informed choice. Such conditions may encompass social, economic, institutional, political, geographical, and structural factors that shape the opportunities and constraints surrounding a choice [11, 12]. These may include, for example, the regulatory design of the choice system, the availability and accessibility of alternative providers, the visibility of differences between providers, and the economic or social consequences associated with choosing a particular provider.

Few studies have examined patients’ understandings of the contextual conditions surrounding the choice of a health care provider. Those studies are often performed in hospital settings and are limited to certain sub-groups of patients, such as patients undergoing cancer treatment. The results show that patients are generally not inclined to actively seek information before making a choice, often because there are no alternative hospitals within a reasonable distance, they cannot observe meaningful differences in quality, or they believe they can simply switch providers if dissatisfied [21–23]. While these findings suggest that contextual conditions affect patients’ engagement in informed decision-making, research remains limited and warrants further investigation. Additionally, previous studies have not addressed how these contextual conditions impact patients’ engagement in choosing a primary care provider, the first point of contact for most healthcare needs.

Of specific concern in this study is therefore patients’ rationalizations and articulations of their approaches choosing a primary care provider and how these approaches are linked to their understandings of, for instance, the institutional, economic and social setting of the provided services. The study approaches the task with the assumption that the contextual conditions of choice systems vary depending on the types of services provided, and that these conditions become more apparent when different contexts of choice systems are compared. This implies the need to contrast patients’ choices of healthcare providers with choices related to other services, such as the choice of an educational institution or a nursing home, to better comprehend their understandings of the specific contextual conditions surrounding the choice of a primary care provider. Accordingly, this study investigates the following research questions:

Compared to decisions in other contexts:

1. to what extent are patients engaged in the informed choice of primary care provider?
2. how do patients understand the contextual conditions surrounding the choice of primary care provider, and how may this understanding influence their information-seeking practices?

### Study setting

The study was conducted in Sweden, a suitable setting for this study for several reasons. Firstly, Swedish patients have extensive statutory rights to choose a primary care provider. The statutory right to choose a primary care provider was introduced nationally in 2010. Although the provision and financing of healthcare services in Sweden are highly decentralized across 21 self-governing regions, legislation required all regions to organize primary healthcare according to a patient choice system [24]. In 2015, patients’ right to choose healthcare providers was further extended to publicly funded outpatient care throughout Sweden, including outpatient specialist care [25]. Through the choice system, Swedish patients are expected to signal to primary care providers to improve quality, based on their informed choice. Implicitly, this suggests that underperforming providers should be outcompeted by not being chosen [26]. The choice system has the characteristics of a quasi-market, meaning that private providers of primary care enter the market through public procurement and compete side by side with public providers. To stimulate competition a larger share of the reimbursement to providers follows patients’ choices. Yet, following the principles of the quasi-market, patients do not purchase primary care services at their own expense at a price determined by supply and demand, but with public funding at fixed prices [27]. Only a small share is financed by patient fees (about 30€ per visit) [28]. Thus, patients may, free of charge and throughout the country, choose which primary care provider to seek care from.

Since the completion of data collection, the regulatory context surrounding patient choice of primary care providers has evolved. In 2022, a listing system was introduced, formalizing patients’ affiliation with a chosen primary care provider and the process for changing this affiliation [29]. However, patients’ general right to seek publicly funded outpatient care from other providers remained unchanged. Thus, the reform formalized the choice of primary care provider without substantially restricting patients’ ability to seek care elsewhere.

The information infrastructure supporting informed choice has also evolved over time. A national digital comparison tool available before 2019 was discontinued around the time of data collection in 2020. Basic provider information, such as location, opening hours, and services offered, remained available. Quality information was also available, including measures of patient-reported experiences, accessibility and waiting times, as well as clinical and process-related quality indicators [30]. However, the opportunities to readily compare such information across providers were more limited during this period. A national comparison tool was reintroduced in 2025 [31].

The statutory patient choice system in primary care primarily concerns the choice of and listing with a primary health care center (PHCC). These are larger health care units, usually staffed with multi-disciplinary teams consisting of 4–5 physicians, registered nurses, midwives and sometimes also physiotherapists and psychologists [28]. From here on, primary care providers will therefore be referred to as PHCCs.

Secondly, Sweden is also a suitable setting for this study as citizens may choose providers of many other types of services. The context of this facilitated the possibility to contrast understandings of an informed choice of a PHCC to that of other types of choices. Similar to the primary care choice system, other public services, such as preschools, schools, and nursing homes, are also organized into a choice system with the purpose of stimulating active choices and competition [32–34]. In addition, several services in the Swedish infrastructural field are deregulated to allow for consumers to freely choose between, for instance, mobile operators and electricity suppliers [35].

## Methods

### Study design

The overall aim of this study is to advance understanding of why patients show limited involvement in making informed choices about health care providers, specifically when choosing a PHCC. However, contextual conditions are not deterministic and to gain a more nuanced understanding of how these conditions influence patients’ behavior, one must consider patients’ interpretations and perceptions [36]. This was performed by using a qualitative approach based on inductive thematic analysis [37] of interviews with a total of 18 respondents. A study with a different focus based on the same interview data has previously been published [38].

To distinguish patients’ understandings of the specific contextual conditions of the choice of a PHCC and how those conditions may influence their inclination to make an informed choice, respondents were invited to contrast their choice of a primary care provider to the process of choosing providers for other services. Throughout the interviews respondents were invited to rationalize their choice of a PHCC. Moreover, they were asked to describe how they had performed their choice of a PHCC with respect to other choice situations and what factors had led them to take potentially different measures to arrive at their choice. As respondents could freely indicate which choice of providers of other services they had found important or had experience of, the examples concerned everything from mobile operators to pre-schools. This enabled the identification of a broad set of contextual conditions which, depending on their prevalence in various choice settings, could either motivate or demotivate respondents to make informed decisions.

### Sample and data collection

The data set was collected through purposeful sampling and based on the principle of maximal variation [39, 40]. This resulted in respondents with a broad range of backgrounds and characteristics including age, gender, origin, occupation and place of residence. For a more detailed description of the respondents’ background characteristics see Table 1.

**Table 1:** Background characteristics of respondents (*n* = 18)

	Numbers
**Gender**	
Female male	9 9
**Age**	
20–30 31–40 41–50 51–60 61–70 71–80	4 2 4 3 3 2
**Place of living**	
Urban Rural	14 4
**Level of education**	
Elementary School Upper Secondary School University studies	1 9 8
**Employment**	
Full time/part time employed Entrepreneur Student On sick leave On parental leave Pensioner	7 2 2 2 1 4
**Country of birth**	
Sweden Other European country Outside Europe	14 0 4

The interviews were performed during the period of June to November 2020, in a large city in mid-Sweden corresponding to both rural and more densely populated areas. Respondents were mainly recruited through outreach to various civic settings, including workplaces, cultural activity centers, nursing homes, trade unions and sports clubs. The civic institutions were asked to distribute an information letter about the study to eligible respondents, along with an invitation to participate. If the contacted respondents were interested in participating, they were then reached by phone or email by the researcher responsible for conducting the interviews. In line with snowball sampling, some respondents were also recruited based on recommendations from the initial respondents.

All the sample respondents received written information about the study and were asked to complete a consent form. At the point of the interview, respondents were also given oral information about the study and their right to withdraw from further participation at any time.

The criteria for inclusion in the study were as follows: age (18+), registered as living in Sweden, and experience of seeking care at a PHCC. At the time of the study, only individuals who were of legal age and registered residents in Sweden were allowed to choose a PHCC. Also, for the purpose of the study, experience of seeking care at a PHCC was vital to be able to reason and explain under what circumstances a choice was made. Consequently, exclusion criteria were minors, nonregistered residents in Sweden and people without experience from seeking care at a PHCC. The collection of respondents continued until data saturation was met, meaning that no more perspectives on the phenomena studied could be found [41].

An interview protocol with semi-structured questions was used to conduct the interviews. Examples of questions were to what extent respondents felt it was important to actively choose a PHCC compared to other types of choices. Other examples of questions were how respondents approached their most recent important choice, followed by how they went about choosing their PHCC. Respondents were also asked to answer some questions about their background, such as date of birth, place of birth, and their self-reported health. The protocol was scrutinized by the research team to which the authors of this study belonged and was pilot-tested to assess face validity. Interviews were performed on Zoom (*n* = 11), by telephone (*n* = 6), and by face-to-face (*n* = 1) and lasted on average about 55 min. One of the interviews was conducted with the support of an interpreter. All interviews were conducted, recorded and transcribed verbatim by the main author of this study.

### Analysis

Thematic analysis was chosen as the analytical approach because it was well suited to the study’s exploratory aim to advance understanding of why patients show limited involvement in making informed choices about PHCCs. Specifically, the analysis focused on how patients perceive and interpret the contextual conditions surrounding the choice of a PHCC, including in comparison with choices in other contexts. Thematic analysis enabled the identification of patterns and variations across participants’ accounts while retaining the contextual and experiential dimensions of their responses.

The analysis was informed by Braun and Clarke’s [37] approach to thematic analysis. First, two authors read the transcripts in full to familiarize themselves with the dataset. Second, the first author identified text segments relevant to the study aim and reviewed this selection twice to minimize the risk of overlooking relevant information. The selected segments were summarized into condensed meaning units and coded according to their content. Similar meaning units were assigned the same code. An example of the coding process is presented in Table 2.

**Table 2:** Example of the analytical process from meaning unit to theme

Informant	Meaning unit	Condensed meaning unit	Code	Theme
Respondent 14, pp. 222, 10,568–10,579	Interviewer: I’m curious—was there a reason you didn’t feel the need to investigate the new Primary Health Care Center before switching? Respondent: Well, I’ve been thinking that if it shouldn’t be good, I would be able to switch again, because there was that opportunity by then, it was like that, when they opened for that (the patient choice reform).(…).	You can switch Primary Health Care Center if you are not satisfied with the quality.	Low switching-costs	The degree of continuity in the relationship with the provider

The initial coding was conducted by the first author and subsequently discussed and reviewed with one co-author over several rounds of analysis. Codes were organized into preliminary themes, which were reviewed against the coded meaning units and the dataset. The coding scheme and preliminary themes were iteratively revised, with the first author and one co-author jointly reviewing the meaning units, codes, and themes and resolving uncertainties through discussion.

All authors were involved in the subsequent interpretation and development of the themes, discussing and reviewing them in relation to the codes and underlying data. Through this collaborative process, the themes were further refined, defined, and named to ensure a coherent structure and clear distinctions between themes. Some themes were merged or separated during this process. Finally, the themes were developed into a coherent narrative and substantiated with illustrative examples from the coded text extracts.

## Results

### An overall low engagement in the choice of a PHCC

The first main finding in the analysis of interviews was that respondents indicated an overall low engagement in the choice of a PHCC. Few had spent significant time thinking through the various alternatives of PHCCs or searching for information about their services and clinical performance. Most respondents had only considered one provider prior to choice and relied almost exclusively on family members’ or friends’ recommendations of which provider to choose. In other choice situations, such as choosing a school, nursing home, or mobile operator, respondents described being more engaged in the choice process. In these other cases, they had invested time in both considering and comparing different alternatives of providers, searching for different types of information and from a wider range of information sources to optimize their choice.

### Five significant differences in contextual conditions

A second main finding was the differences in how respondents comprehended the contextual conditions surrounding the choice of a PHCC with respect to other choice situations. Five themes of contextual conditions emerged in the analysis of the interviews. Furthermore, respondents’ perception of the degree of occurrence of each contextual condition seemed to influence respondents’ engagement in the choice of provider. The five themes are displayed in Table 3 and summarize respondents’ understanding of the occurrence of each condition of the choice of a PHCC in relation to other types of choices. In the following sections a thorough description of each theme is presented.

**Table 3:** Five themes describing respondents’ understandings of the contextual conditions for choice in different settings

Contextual condition	Choice of PHCC	Other choices
The perceived necessity to choose	Low	High
The visibility of differences between providers	Low	High
The expectation of equal service quality	High	Low
The presence of personal relationships connected to providers	Low	High
The continuity of the care relationship	Low	High

#### Theme 1: The perceived necessity to choose

The first theme describes how the presence or absence of the imperative to choose a provider can either motivate or discourage an informed choice. The analysis of interviews revealed that respondents held varying beliefs regarding the importance of choosing a PHCC compared to selecting a provider in other settings. With respect to the choice of a PHCC, respondents did not perceive the choice as especially necessary. This was, for instance, illustrated by respondents who felt the authorities did not actively prompt them to choose a PHCC when it was relevant to enroll with a provider. Respondents also argued they could access primary care services without actively choosing to enroll with a specific PHCC. “(…) it’s not like they denied me care; they have received me anyway”. (Respondent 4). Furthermore, there was a belief that one could receive similar care without even turning to a PHCC. Respondents referred, for instance, to seeking care at the local emergency ward or using private health care insurance, which they argued made it less relevant to engage in the choice of a specific PHCC.

Contrary, respondents described the choice in other situations as more necessary to make. This was especially evident for decisions concerning schools or nursing homes. Respondents described how the authorities had urged them to make an active choice either through an information letter sent to their household or through verbal communication. Furthermore, they were asked to rank several alternatives of schools and nursing homes since the authorities could not guarantee they would be assigned their first option. “(…) yes you choose, I think it’s nine alternatives (pre-schools) or something in this municipality”. (Respondent 11). Hence, in these instances respondents felt decision-making was necessary to perform to access the services in the first place.

#### Theme 2: The visibility of differences between providers

The second theme emphasizes how the visibility of differences in services may moderate the inclination to make an informed choice. Respondents expressed that an important rational for making an informed choice of a PHCC would be differentials in the range and quality of services. For example, respondents cited care tailored to older patients or shorter waiting times. Yet, there was an overall sentiment that the PHCCs offered more or less the same services, which made respondents think it was excessive to actively compare and choose a specific provider over another. This belief was reinforced by respondents’ impression that the providers did not actively promote their services to attract more patients and that available information describing the differences was scarce. “I haven’t performed any large-scale seeking, but it’s nothing that I found that these PHCC’s like try to promote themselves”. (Respondent 4)

In other choice contexts respondents described how the differences in the services offered were much more noticeable and actively promoted by both authorities and providers, something which made them more motivated to compare and seek information about different options.

*For instance*,* you can read that one educator has 15 pupils*,* or ‘we have this pedagogy’*,* or here you need to study a language and then a language trip is included*,* it’s more selling of one’s businesses in a sense. (Respondent 4).*

Besides being actively invited to take part in publicly disclosed comparative information about the different options of, for instance, pre-schools or nursing homes, respondents also came in touch with marketing materials issued by the providers themselves. Not least, this was evident in the choice of schools wherein respondents described how they had been invited to high school fairs and “open houses” where teachers and pupils promoted their services and the nature of the education offered.

#### Theme 3: The expectation of equal service quality

The perception of the extent to which quality and range of services are equitably provided constituted a third theme, which appeared to influence the level of engagement in making an informed choice of a PHCC. For the choice of a PHCC respondents perceived that they would receive health care services of equal quality independent of which PHCC they chose. Partly, this was based on the opinion that providers would charge the same low fee for services regardless of which care facility they visited and, hence, that access to care was not dependent on their ability to pay for the services. Partly, it was based on the opinion that the organizational conditions of the PHCC would not influence the quality of care to such an extent that it would be worth the efforts to make an informed and active choice. Instead, respondents seemed to rely on public authorities to monitor the quality to ensure a minimum standard of services and intervene in the case of inferior quality.

*(…) no I just feel that they are relatively equal*,* maybe I trust the Swedish system*,* the welfare society (…) in this case I believe existing monitoring authorities and so on works. (Respondent 17).*

Likewise, the medical profession was regarded as highly capable to provide equal medical treatment. This was exemplified by respondents who questioned why doctors would achieve different clinical results, and which seemed to further reinforce the opinion that seeking information about the PHCC’ clinical performance or the competence of the staff was not especially relevant. Yet, there were exceptions. A few respondents had invested time in their choice through, for instance, considering and comparing several primary care providers based on the clinical experience of doctors or management on the part of the providers. Hence, contrary to the general picture, some respondents expressed the importance of an informed choice of a PHCC due to the lack of trust that providers or individual doctors would offer care of equal quality.

In the case of other choice situations, respondents perceived neither the pricing nor the level of quality of providers’ services as equal. For instance, respondents described how they had put a lot of effort in comparing the quality and environmental factors of different schools since they experienced large differences in the education being provided.

*I believe that if my son attends a good school*,* he will develop in a completely different way*,* both as a person*,* socially*,* and in terms of his overall knowledge level*,* than if he attends the wrong school. (Respondent 10)*

Respondents also described the importance of being involved in making an informed choice of an educational provider since they believed it would have a larger impact on multiple prospects of their children’s lives. Overall, they did not indicate the belief that the choice of school or education would automatically correlate to positive outcomes but felt the need to invest time in the decision in order to secure certain prospects for their children’s futures.

#### Theme 4: The presence of personal relationships connected to providers

Theme four evolved from respondents’ descriptions of the importance of personal relationships as a key factor influencing the level of active engagement in the informed choice process. In the case of primary care, respondents described they had not put any greater effort into considering or seeking information about the different PHCCs, since they felt the only relationship they had at the PHCC was the relation to their doctor. This was, for instance, illustrated by respondents who had just followed their current doctor to a new PHCC without reflecting upon the quality of services of the new provider or considered other alternatives of providers to choose from.

*No*,* I didn’t find out anything about the PHCC*,* it was just that he (the doctor) informed me ‘now I quit here at this PHCC*,* and we will establish a new Center in another area’*,* and then I just said I wanted to follow along. (Respondent 10)*

Hence, respondents seemed to regard the choice as solely derived from the patient-doctor relationship, as opposed to a constellation of relationships occurring within the PHCC organization. In other types of choices, respondents seemed to be more urged to seek information about the different providers, due to the larger number of relations they would have at the chosen provider.

*Regarding the nursing home or elderly care*,* there I believe (…)*,* like with the number of relations you have*,* you meet many people*,* and I know how important it is – the relation –*,* especially if you’re fragile and so on. (Respondent 2)*

In other types of choices, such as the choice of an educational provider, respondents also seemed to conceive of the choice as a choice of a collective unit than a choice of a personal contact to a specific profession. Respondents described, for instance, how they had invested time in becoming acquainted not only with teachers, but also other pupils and parents at the school to compose a picture of the school environment and future social networks. In this case, the choice seemed to be as much a selection of a marker of social identity as a choice of a provider.

#### Theme 5: The continuity of the care relationship

Finally, theme five illustrated how the continuity of the care relationship was another contextual condition that respondents considered when deciding whether to inform themselves about the various alternatives of PHCCs. Concerning the choice of a PHCC, respondents believed it was unnecessary to dwell into detailed information about the different alternatives since their visits at the PHCC did not last that long. Furthermore, respondents did not seem to experience any greater emotional cost associated with severing ties with the PHCC and argued they could always switch provider if dissatisfied with the offered quality of services. For these reasons respondents seemed to think it did not matter that much if their choice of a PHCC was not optimal from the beginning. “I’ve been thinking that if it shouldn’t be good, I would be able to switch again”. (Respondent 16).

However, there were opposite opinions expressed in the interviews. One respondent described the importance of making the right choice from the beginning to not be disappointed with the services being offered once visiting the clinic. There were also respondents who spent more time at the PHCC and, thus, believed it was more important to engage in information-seeking prior to choice.

In other choice situations, it was more evident that respondents felt motivated to engage in informing themselves about the choice as they felt they would have a more sustainable relation with the provider. Again, this was illustrated especially by the choice of an educational provider or a nursing home, where respondents described how they or their close relatives would have to spend several months or even years with the provider in question. Respondents also expressed that they felt it more difficult to break loose and switch to another provider once having established relations to the chosen alternative and its geographical location. One respondent exemplified with the choice of university:

*(…) if it feels wrong (…) I don’t want to move to this other town (…) that will affect my studies. So*,* it’s important to feel comfortable with the site and the environment*,* with everything when I’m studying*,* especially during five years or even more*. *(Respondent 12)*.

There were also respondents who stressed the emotional costs of making the wrong choice, for instance, having their children switch school in the middle of a semester and risk losing secure relations with both teachers and other pupils. This cost, they argued, was much bigger when switching school compared to switching PHCC and had made them put more effort into the choice of the educational provider than the PHCC. Yet, there were also respondents with the opposite opinion who argued they could, for instance, always switch pre-school if they would evaluate the quality as low or if their child did not feel comfortable in the setting.

## Discussion

The overall aim of this study was to advance understanding of why patients show limited involvement in making informed choices about health care providers, specifically when choosing a PHCC. This was addressed by examining how patients, in comparison with other choice contexts, understand the contextual conditions surrounding the choice and how these understandings may affect their inclination to search for and make use of information.

Several respondents in this study indicated that they had actively chosen a PHCC. Yet, the overall trend indicated limited involvement in the decision-making process. Although being legally entitled to choose PHCCs, respondents described a sense of “business as usual” in how they navigated through the system. In accordance with earlier studies [7, 10] few of the respondents had considered several alternatives or searched for information about the providers prior to their decision. This less engaged approach to choosing distinguishes itself from other types of choices, such as choosing a school, a nursing home or a mobile operator. In these situations, respondents described a more deliberate approach to choosing as they had both invested more time in considering different alternatives and searched for information prior to their choice.

The results of this study indicated that respondents’ understanding of the contextual conditions of choosing a PHCC influenced how they engaged in the choice. Related to other types of choices those contextual conditions became even more distinct. In line with previous studies, we found that patients had limited incentives to make an informed choice of a PHCC when they perceived the level of quality as equal or found that switching costs were low if dissatisfied [21, 23].

Yet, results also revealed other contextual conditions that reduced patients’ motivation to seek information before choosing a primary care provider. Unlike choices such as selecting a school, respondents felt little need to commit to a specific PHCC, knowing they could access care elsewhere, like at local emergency clinics. They also perceived fewer differences between PHCCs, noting a lack of promotion or branding. Social factors, important in other contexts like education, played a minimal role, as respondents were more attached to individual doctors than to the PHCC itself. The finding that respondents were more attached to individual physicians than to the PHCC as an organisation may also be understood in relation to physician loyalty. Raj and Banaszak-Holl [42] identify loyalty to physicians as a potentially important mechanism influencing provider choice, while noting that it remains insufficiently explored in the literature on consumer engagement with performance information. Such loyalty may reduce the perceived relevance of comparative information about PHCCs, particularly when patients already have an established relationship with a trusted physician. In this sense, patients’ engagement with informed choice may not only depend on their ability or willingness to process information, but also on the relational context in which the choice is made.

The results of this study emphasize the importance of considering how the contextual conditions surrounding the choice shape the way patients understand the necessity to engage in the informed choice of a health care provider. As shown in the literature review, earlier studies have mostly turned to internal cognitive abilities in the individual in trying to explain their difficulties or unconcern about considering different health care providers based on clinical performance information [15, 18]. However, earlier studies appear to remain influenced by a rationalist paradigm in how they view patients’ use of information—assuming that patients are both willing and able to seek out large amounts of information before making a choice – if only the information were better designed and more easily accessible. Our results demonstrate that patients’ remote approach to the informed choice of primary care provider is not only a matter of cognitive challenges shaped from within the individual, but also a matter of the external, contextual conditions surrounding patients’ choices. This interpretation is supported by experimental evidence from Swedish primary care. Anell et al. [43] found that providing consumers with comparative information and reducing practical barriers to switching increased the propensity to change provider, demonstrating that information and switching costs can influence provider choice. However, the relatively modest effects in the general population also suggest that providing information alone may not be sufficient to generate active engagement with provider choice. Contrary to rational choice theory [44], our results acknowledge that patients’ choice of a PHCC is not for most a product of a cost-benefit analysis, but a result of, for instance, the way in which they perceive that the institutional setting promotes the need to actively make a choice or the type of social relation they have to the provider. Consequently, the results challenge the dominant rationale behind several governments’ decisions to introduce choice systems in health care – not least in Sweden [26].

The results also suggest that the degree of marketization influences individuals’ inclination to make informed choices. Perhaps unsurprisingly, respondents actively sought information when choosing private providers such as mobile or solar cell companies, due to clear differences in price and quality.

More unexpectedly, there were notable contrasts between the choice of a PHCC and other public services. For example, the Swedish school system appeared more marketized, with provider competition, active promotion, and publicly available comparative data prompting more information-seeking. Respondents also assumed significant variation in school quality, which increased their motivation to engage. In contrast, respondents expected similar standards across PHCCs and observed little marketing. These findings are consistent with Vengberg [45], who found that few PHCCs aimed to expand, relying instead on a steady inflow of patients.

### Methodological considerations

The findings of this study should be interpreted in relation to the institutional context prevailing in 2020, when the statutory right to choose a primary care provider was already established. Subsequent legislative changes in 2022 formalized the procedure for choosing and being listed with a provider, without restricting patients’ general right to seek care from other providers. The digital infrastructure supporting choice has also developed, with new tools enabling patients to compare different providers. It is not, however, evident that institutional changes of mainly administrative character imply corresponding changes in how individuals perceive or engage with choice, making this an important area for future research.

Although the study included 18 participants, the sample size was considered appropriate given the narrowly defined research focus and the use of maximum variation sampling to capture diverse perspectives. Previous empirical research suggests that relatively small samples can be sufficient to reach saturation in qualitative studies with focused research questions [46]. In this study, interviews continued until subsequent interviews largely repeated information from earlier interviews, suggesting that saturation had been reached. The sample was therefore not intended to achieve statistical representativeness, but to capture variation in experiences and perspectives relevant to the research question. Furthermore, the study sample consisted of a diverse group of respondents with varying backgrounds, encompassing both demographic and socioeconomic characteristics. This diversity improved the possibilities of achieving a rich picture on the phenomenon studied [41]. Yet, there might have been a bias of certain perspectives, especially due to the overrepresentation of respondents with longer educational backgrounds and urban residents.

Due to data being collected during the pandemic and the necessity to handle it safely, interviews were almost exclusively conducted by telephone or zoom. Earlier studies reported no significant differences in results by using different interview modes [47, 48]. Yet, interviews conducted in person, where important bodies and facial expressions are more visible, could have facilitated further probing and, consequently, elicited more nuanced and detailed answers.

During the interviews, respondents were not explicitly asked to recall how much time had elapsed since they chose their primary care provider or other service providers. This allowed respondents to freely elaborate on their perceptions and reasoning concerning primary care choice, rather than being constrained to reconstructing the precise circumstances and details of a specific past choice. However, it should be acknowledged that a longer interval between the choice and the interview may have increased the risk of recall bias, particularly regarding the specific process through which the choice was made.

The rigor of study results was attained by using researcher triangulation. Two of the authors read through the entire dataset, discussed, and revised coding and themes during the analysis. All authors were involved in scrutinizing the coding scheme and themes to enhance the confirmability of the findings. Also, authors used illustrative quotations from respondents to further reinforce the confirmability of results.

Last, although some of the results were in line with earlier studies, and thus provided some evidence of external consistency, the Swedish study setting may have limited the transferability of the findings [49]. For instance, the design of the choice systems may differ from those in other countries and consequently may have affected the way in which respondents perceived the structural conditions to carry out an informed choice.

## Conclusions

Overall, patients showed limited involvement in making an informed choice of primary care provider. Compared to the decision process in many other choice situations, they had neither considered other alternatives nor searched for such information that could underpin a reasonable choice. Hence, results thus question one of the most fundamental principles of patient choice reforms, namely that patients’ choices will promote increased quality of services. Additionally, this study highlights how external, contextual conditions may shape patients’ engagement in making an informed choice of a primary care provider.

From a policy perspective, results demonstrate both the opportunities and difficulties in creating a market-like situation in primary care. Patients might be more inclined to make an informed choice of a primary care provider if the choice was perceived as more urgent and if providers were incentivized to differentiate their services, promoting distinctions in clinical quality. Conversely, our findings suggest that greater efforts by authorities and providers to promote choice or highlight differences in quality may not automatically lead to more engaged and informed choices. Rather, patients’ engagement appeared to be shaped by how they understood the contextual conditions of the choice, including the extent to which they perceived meaningful differences between providers and trusted that comparable quality of care could be expected across providers. Compared to other choice situations, respondents gave proof of a significant trust in the clinical quality of primary care services and even argued it should not differ. Furthermore, they were not, for the most part, concerned about the organizational qualities of the primary care provider per se, but rather about the personal relationship they had with their doctor.

## Data Availability

The data that support the findings of this study are available from the corresponding author, Dr. Caroline Blomberg-Ohlström, upon reasonable request. However, data is subject to privacy restrictions and requires appropriate ethical approval for access.

## Declarations

### Permission to reproduce material from other sources

Not relevant.
